# Concomitant stress potentiates the preference for, and consumption of, ethanol induced by chronic pre-exposure to ethanol

**DOI:** 10.1590/1414-431X20155009

**Published:** 2015-11-27

**Authors:** G. Morais-Silva, J. Fernandes-Santos, D. Moreira-Silva, M.T. Marin

**Affiliations:** 1 Instituto de Ciências Biomédicas, Universidade Federal de Uberlândia, Uberlândia, MG, Brasil; 2 Laboratório de Farmacologia, Departamento de Princípios Ativos Naturais e Toxicologia, Faculdade de Ciências Farmacêuticas, Universidade Estadual Paulista (UNESP), Araraquara, SP, Brasil; 3 Programa Interinstitucional de Pós-Graduação em Ciências Fisiológicas, Universidade Federal de São Carlos/Universidade Estadual Paulista (UFSCar/UNESP), São Carlos, SP, Brasil

**Keywords:** Ethanol, Addiction, Stress, Free-bottle choice, Withdrawal

## Abstract

Ethanol abuse is linked to several acute and chronic injuries that can lead to health problems. Ethanol addiction is one of the most severe diseases linked to the abuse of this drug. Symptoms of ethanol addiction include compulsive substance intake and withdrawal syndrome. Stress exposure has an important role in addictive behavior for many drugs of abuse (including ethanol), but the consequences of stress and ethanol in the organism when these factors are concomitant results in a complex interaction. We investigated the effects of concomitant, chronic administration of ethanol and stress exposure on the withdrawal and consumption of, as well as the preference for, ethanol in mice. Male Swiss mice (30–35 g, 8-10 per group) were exposed to an ethanol liquid diet as the only source of food for 15 days. In the final 5 days, they were exposed to forced swimming stress. Twelve hours after removal of the ethanol liquid diet, animals were evaluated for ethanol withdrawal by measuring anxiety-related behaviors and locomotor activity. Twenty-four hours after evaluation of ethanol withdrawal, they were evaluated for voluntary consumption of ethanol in a “three-bottle choice” paradigm. Mice exposed to chronic consumption of ethanol had decreased locomotor activity during withdrawal. Contrary to our expectations, a concomitant forced swimming stress did not aggravate ethanol withdrawal. Nevertheless, simultaneous ethanol administration and stress exposure increased voluntary consumption of ethanol, mainly solutions containing high concentrations of ethanol. These results showed that stressful situations during ethanol intake may aggravate specific addiction-related behaviors.

## Introduction

Alcohol addiction can be conceptualized as a set of cognitive, behavioral and physiologic symptoms that indicate a loss of control of substance use by individuals, along with continued use of the substance despite adverse consequences. Symptoms of ethanol addiction include compulsive substance intake and withdrawal syndrome (negative emotional and physiologic states that appear after cessation of substance use) (1).

Several animal models have been developed to study alcohol addiction. In general, they involve forced exposure of animals to alcohol for prolonged periods. A useful model to induce behavioral and neurologic changes related to addiction is forced consumption of ethanol as the only source of food in a liquid diet for several weeks. Rodents exposed to this regimen show behavioral and neural alterations related to ethanol use (2).

Changes in patterns of ethanol consumption are also features of alcoholism. Animals treated chronically with ethanol previously show increased consumption of ethanol in a “two-bottle choice” paradigm and in operant procedures (3 4 5).

Individual risk factors for the development of abuse and addiction of alcohol are incompletely understood. Nevertheless, acute and chronic stress have an important role in motivation for the abuse of addictive drugs (6). For example, negative mood states alone appear to be sufficient to elicit a desire for alcohol in humans (7). Personal experience of stress also induces alcohol craving in alcoholics, and is a risk factor for relapse (8). Stressed animals consume more alcohol solutions in a two-bottle choice paradigm (9,10). Mice that have lower preferences for ethanol consume more ethanol if exposed to restraint stress (11).

In general, chronic administration of ethanol or stress exposure enhances addiction-like behaviors. However, if these two factors are present simultaneously, the interaction becomes complex. Alcohol has anxiety-reducing properties and can relieve stress but acts simultaneously as a stressor and activates stress-response systems (12). Alcohol gavage blocks stress-induced impairments in memory and anxiety (13). Ethanol injection before immobilization stress also reverses some stress-induced changes in catecholamine levels within the brain (14).

We investigated the effects of concomitant, chronic administration of ethanol and stress exposure upon the withdrawal and consumption of, as well as the preferences for, ethanol in mice.

## Material and Methods

### Subjects

The experimental protocol was approved by the Ethics Committee for Animal Utilization of Universidade Federal de Uberlândia (CEUA 120/11). Experiments were conducted according to the principles of the Brazilian College of Animal Experimentation, which are based on the Guidelines for the Care and Use of Laboratory Animals (NIH, Bethesda, MD, USA).

Male Swiss mice (Vallée Institute, Uberlândia, MG, Brazil; 30-35 g) were transferred to our animal facility ≥5 days before the start of experimentation and housed in groups of 4-5 per cage. The room was maintained at 23±2°C on a 12-h light-dark cycle with access to water and food *ad libitum*(except during the liquid-diet procedure). Experiments were carried out during the light phase of the cycle, and animals were tested randomly across this time period (8–10 mice per group).

### Chronic administration of ethanol

The procedure for chronic administration of ethanol was adapted from experiments with intake of a forced liquid diet undertaken by Bonassoli et al. (15). The liquid-diet procedure was chosen over other methods because it is operationally easier and causes less stress to animals.

Animals allocated in plastic cages [19 (width)×30 (length)×13 cm (height), 4-5 animals per cage] had free access to bottles containing a solution composed of Sustagen M¯ (chocolate flavor; Mead Johnson, Brazil) at 28.5 g/100 mL (1.1 kcal/mL) and no chow. The liquid diet was the only source of food available to mice; it was prepared fresh every day and presented to animals at the same time (12 pm). This liquid diet provided all necessary nutrients for rodents and was presented in a larger volume than normally eaten to ensure consumption *ad libitum*. Bottles were weighed before and after exposure to mice to evaluate diet consumption per animal group; any remaining food was removed after 24-h exposure. One bottle of drinking water was available simultaneously with the liquid diet. Mice were kept in groups with no restricted volumes of the diet to reduce the stress related to isolation or food restriction.

**Figure 1:**
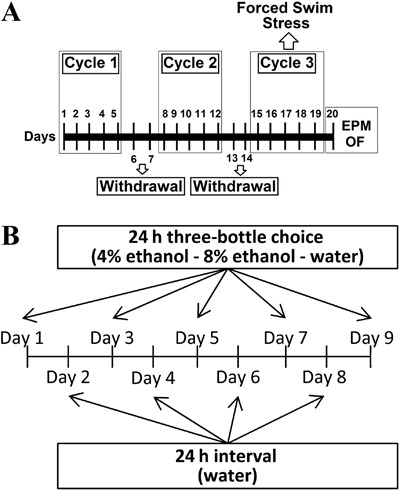
*A*, Procedural timeline for chronic administration of ethanol and stress testing. *B*, Experimental protocol used for voluntary consumption of ethanol. EPM: elevated plus maze; OF: open field.

Liquid diet was administered for 15 days. It was distributed in three cycles of 5 days, with a 2-day interval without the diet (when mice received conventional chow) between each diet cycle. This procedure mimics a kindling mechanism in alcohol withdrawal in which repeated experiences of withdrawal intensify its symptoms (16). Groups treated with ethanol had ethanol 6% (v/v) added to their solution on the first 2 days and ethanol 8% (v/v) on the remaining 13 days, whereas the control group received the same liquid diet without ethanol throughout the experiment (i.e., vehicle). Figure 1A shows the timeline of the experimental procedure. Body weight, liquid diet solution, and ethanol consumed over the study are shown in Table 1. Importantly, during chronic administration of the diet, mice were housed in groups. Liquid diet solution and ethanol consumption during this period is an estimate and cannot reflect group differences in feeding.

**Table:**
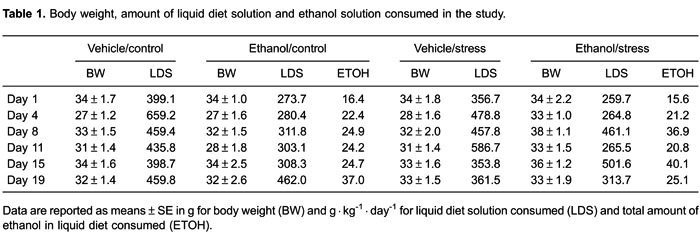

### Stress

Over 5 consecutive days, mice from the stress group were placed in a cylindrical 5-L container [22 (height)×17 cm (diameter)] filled with 3.5 L of water, from which mice could not escape. On days 1, 3, and 5, mice remained in the water for a single trial of 15 min. On days 2 and 4, mice underwent three trials of 6 min with intervals of 5 min. After each swimming session, mice were towel-dried and returned to their home cages. Stress procedures were undertaken at different schedules (9 am to 5 pm). The control group comprised mice not exposed to stress.

### Evaluation of ethanol withdrawal

Ethanol withdrawal was evaluated by behavioral analyses in the elevated plus maze (EPM) and open field (OF) for 12 h and 14 h, respectively, after final exposure to the liquid diet. All behavioral tests were conducted between 9 am to 12 pm.

The EPM comprises a plus-shaped wooden apparatus elevated 38.5 cm above floor level with two open arms [30 cm (length)×5 cm (width)×0.25 cm (height)] and two closed arms [30 cm (length)×5 cm (width)×15 cm (height)] connected by a common central platform [5 cm (length)×5 cm (width)].

Mice were placed in the central platform of the EPM facing an open arm and had their behavior video-recorded by a camera fixed to the roof for 5 min for analyses of the: number of entries (arm entry = 4 paws in the arm) in closed arms; percentage of entries in open arms [(open/total)×100]; percentage of time spent in open arms [(time open/total)×100)]; number of head dips (exploratory movement of the head over the side of the maze); stretched attend postures (exploratory movement in which the body is stretched forward and then retracted to the original position). Behavioral analyses were carried out using the X-Plo-Rat 2005 software (developed by the research team of Dr. Morato, Faculdade de Filosofia, Ciências e Letras de Ribeirão Preto, USP, Brazil).

The OF is a circular arena (Insight Ltd., Brazil), 30 cm in diameter, surrounded by transparent walls (height, 30 cm) with its circular floor divided by lines into four central and eight peripheral quadrants of equivalent area.

Mice were placed in the middle of the apparatus and the percentage of time spent in the center ((time in center/total)×100), time spent in each central quadrant (time in center/number of central quadrants) and number of quadrants crossed was quantified over 5 min for assessment of peripheral, central and total locomotion using the OpenFLD software (developed by Stéfano Pupe Johan, Brazil).

### Voluntary consumption of ethanol

Voluntary consumption of ethanol was evaluated using the “three-bottle choice” paradigm 24 h after evaluation of ethanol withdrawal as described above.

Every other day, for 9 days, mice housed individually in home cages [19 (width)×30 (length)×13 cm (height)] were exposed to three bottles simultaneously: one containing fresh water, one containing ethanol 4% in fresh water (v/v) and another containing ethanol 8% in fresh water (v/v). Every day the positions of the bottles were interchanged, with no preference given to the side of placement. Only fresh water was available during the days that consumption was not measured. Food was available *ad libitum*during this experiment. Bottles were presented to mice at the same time each day (12 pm).

Amount of liquid lost by evaporation or leakage was measured *via*bottles placed in empty cages. Amount of liquid lost in these empty cages was subtracted from the respective solution exposed to mice. Values of ethanol consumption are reported relative to the body weight of mice (g/kg), in preference to ethanol [(total amount of ethanol solution consumed/total amount of fluid)×100] and in preference to bottles containing 4% or 8% of ethanol [(total amount of ethanol 4% or 8% consumed/total amount of fluid)×100]. Figure 1B shows the timeline of the experimental procedure.

### Statistical analyses

Statistical analyses were done using the Statistica software (StatSoft Inc., USA). Data are reported as means±SE of 8-10 animals per group. Results of EPM and OF experiments were analyzed by two-way analysis of variance (ANOVA) considering the factors “chronic ethanol” (ethanol×vehicle) and “stress” (stress×control). Voluntary consumption of ethanol and body weight were analyzed by three-way ANOVA considering the factors “chronic ethanol”, “stress” and “time”. If ANOVA showed significant differences (P≤0.05), a planned comparison test between the groups of interest was undertaken.

## Results

Body weight of mice in groups did not differ from each other throughout the experimental procedure (Table 1). Two-way ANOVA revealed only a significant effect of time (F_5,160_=7.68, P≤0.05).

**Figure 2:**
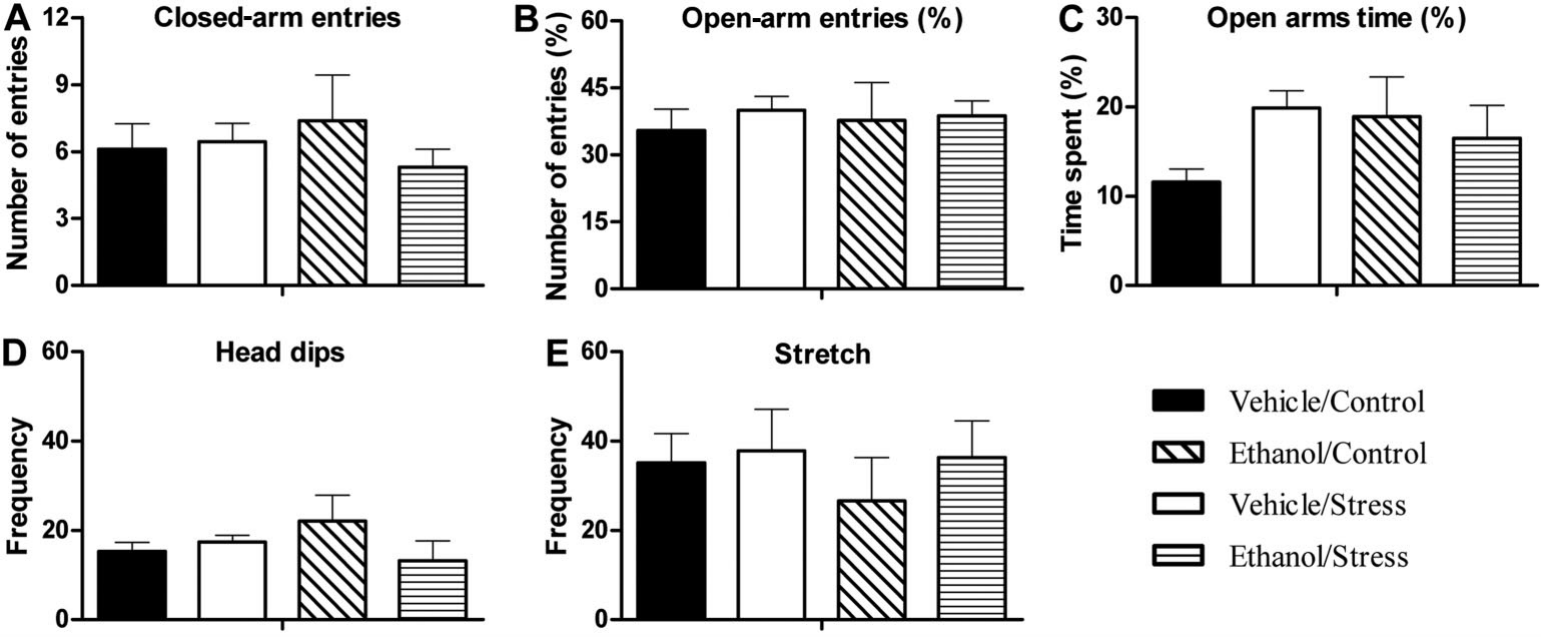
Spatial and ethologic behaviors of mice exposed to the elevated plus maze (EPM) after chronic administration of ethanol and stress testing. Mice were tested for 5 min after 12 h of ethanol withdrawal. Data are reported as means±SE. *A*, Number of closed-arm entries; *B*, percentage of open-arm entries; *C*, percentage of time spent in open arms; *D*, number of head dips; *E*, number of stretch-attend postures. No significant differences were found for mouse behavior in EPM for n=8-10 animals per group.

No significant differences were found for mouse behavior in EPM (Figure 2). All groups had similar values of closed-arm entries, open-arm entries and time, head-dip frequency, and number of stretching postures.

**Figure 3:**
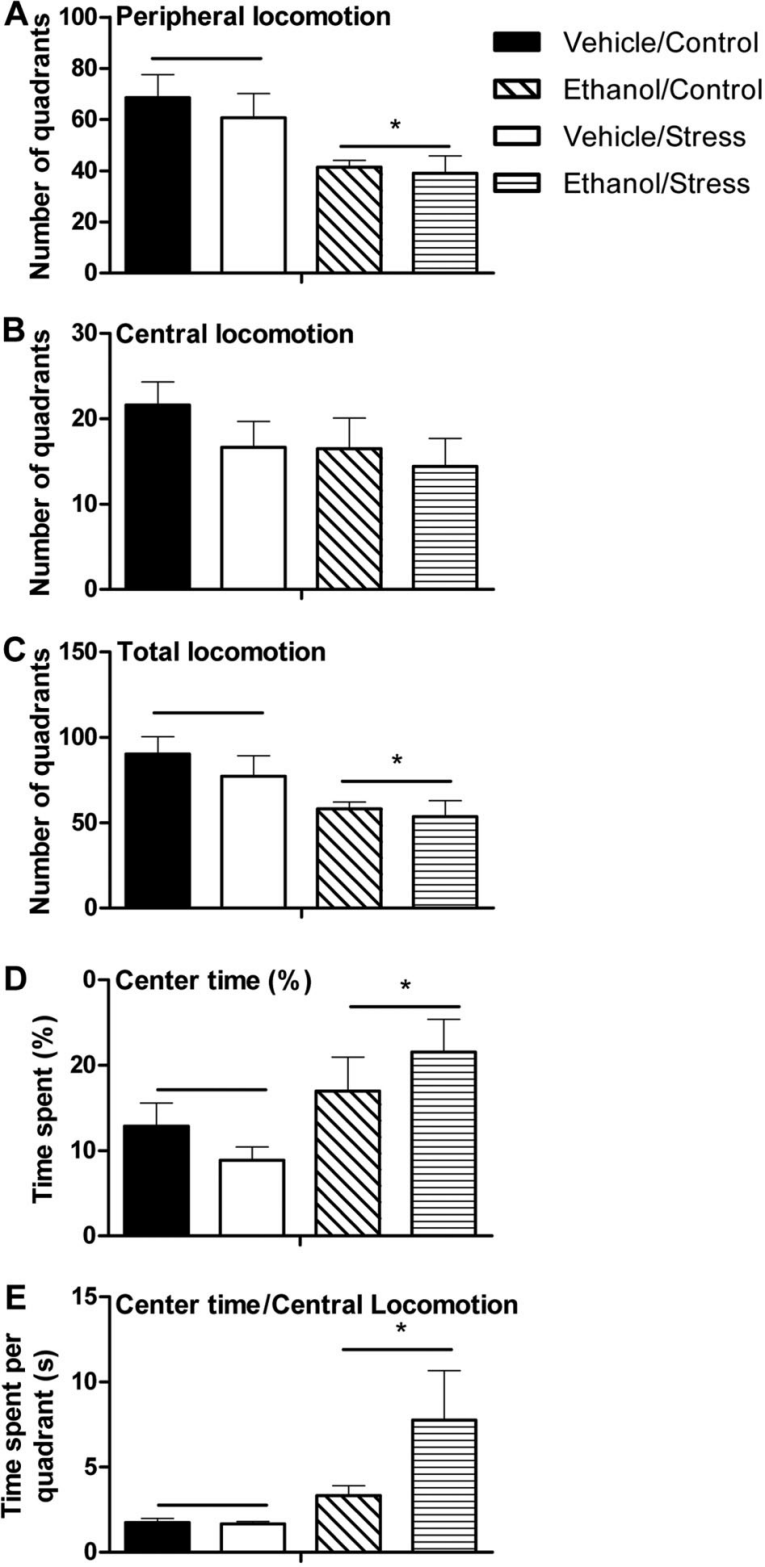
Locomotor activity of mice exposed to the open field apparatus after chronic administration of ethanol and stress testing. Mice were tested for 5 min after 14 h of ethanol withdrawal. Data are reported as means±SE. *A*, Number of quadrants travelled in the periphery of the apparatus; *B*, number of quadrants travelled in the center of the apparatus; *C*, number of quadrants travelled in the total area of the apparatus; *D*, percentage of time spent in the center of the apparatus; *E*, time spent in each central quadrant. *P<0.05 compared with vehicle-treated groups for n=8-10 animals per group (two-way ANOVA test).

In terms of OF behaviors (Figure 3), two-way ANOVA revealed a significant difference for the chronic ethanol factor (F_1,32_=9.72, P≤0.05) in peripheral locomotion but not for the stress factor or interaction, showing a decrease in peripheral locomotion in those groups (Figure 3A). Two-way ANOVA revealed a significant difference for the chronic ethanol factor (F_1,32_=8.31, P≤0.05) in total locomotion but not for the stress factor or interaction, showing a decrease in total locomotion in those groups (Figure 3C). The percentage of time spent in the center was altered by treatment. Two-way ANOVA showed a significant difference for the chronic ethanol factor (F_1,32_=7.28, P≤0.05). Mice from ethanol-treated groups spent more time in the central part of the apparatus than vehicle-treated groups (Figure 3D). No differences were found in central locomotion (Figure 3B). Animals from ethanol-treated groups spent more time in each central quadrant than vehicle-treated groups (Figure 3E), and two-way ANOVA showed a significant difference for the chronic ethanol factor (F_1,32_=6.69, P≤0.05).

**Figure 4:**
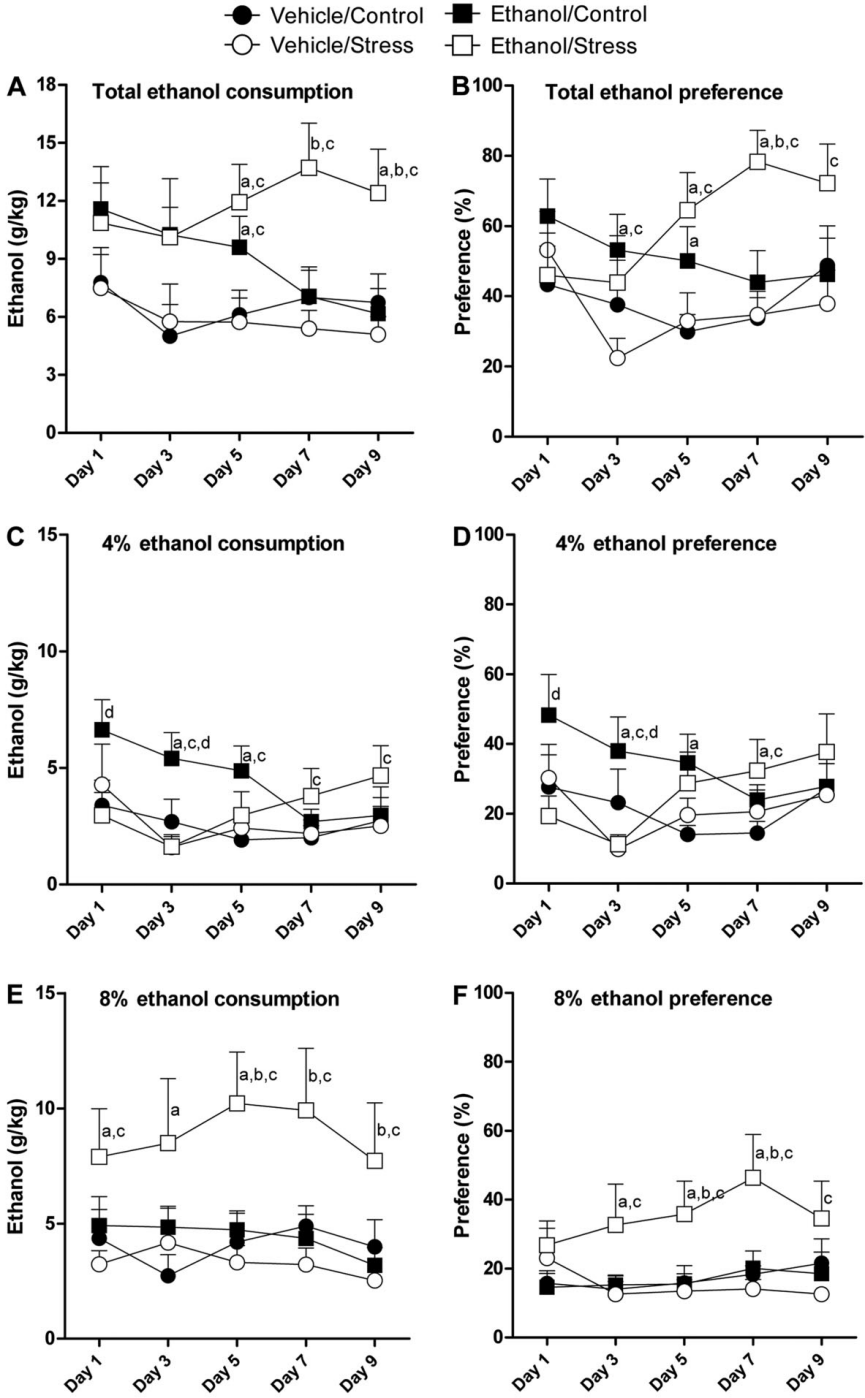
Patterns of voluntary consumption of ethanol during the “three-bottle choice” paradigm. Data are reported as means±SE. *A*, Total ethanol consumption (grams of ethanol per kilogram of animal); *B*, percentage of preference for ethanol solutions; *C*, consumption of 4% ethanol solution; *D*, percentage of preference for 4% ethanol solution; *E,* consumption of 8% ethanol solution; *F*, percentage of preference for 8% ethanol solution. ^a^P<0.05 compared with the vehicle/control group. ^b^P<0.05 compared with the ethanol/control group. ^c^P<0.05 compared with the vehicle/stress group. ^d^P<0.05 compared with the ethanol/stress group for n=8-10 animals per group (three-way ANOVA followed by planned comparisons test).

Figure 4 contains data obtained from assessment of voluntary consumption of ethanol. Total consumption of ethanol was different between groups. Three-way ANOVA showed significant differences for the ethanol factor (F_1,28_=13.63, P≤0.05) and the interaction between ethanol, stress and time factors (F_4,112_=2.79, P≤0.05).

On days 1 and 3, no significant differences were found between experimental groups relating to total consumption of ethanol (P>0.05). On day 5, the ethanol/control group and ethanol/stress group consumed more ethanol compared with the vehicle/control group and vehicle/stress group (P≤0.05). On day 7, the ethanol/control group and ethanol/stress group consumed more ethanol only when compared with the vehicle/stress group (P≤0.05). On day 9, the ethanol/stress group consumed more ethanol when compared with all other groups (P≤0.05).

The total ethanol preference was changed by treatment. Three-way ANOVA showed significant differences for the ethanol factor (F_1,29_=12.15, P≤0.05) and the interaction between ethanol, stress and time factors (F_4,116_=3.78, P≤0.05).

No differences were seen in total ethanol preference on day 1. On day 3, the ethanol/control group had higher preference scores when compared with the vehicle/control group and vehicle/stress group (P≤0.05). On day 5, the total ethanol preference of the ethanol/stress group was significantly greater than the ethanol preference of the vehicle/control group and vehicle/stress group (P≤0.05). On this day, the ethanol/control group had higher preference scores only when compared with the vehicle/control group (P≤0.05). On day 7, the ethanol/stress group had greater preference for ethanol solutions when compared with all other groups (P≤0.05). On day 9, the only difference found in total ethanol preference was between the ethanol/stress group and vehicle/stress group, in which the ethanol/stress group had a higher preference for ethanol solutions.

The ethanol/control group showed increased consumption of 4% ethanol solution. Three-way ANOVA for consumption of 4% ethanol solution showed significant effects for the ethanol factor (F_1,29_=6.85, P≤0.05) and interaction between ethanol, stress and time factors (F_4,112_=2.72, P≤0.05). On day 1, the ethanol/control group consumed more 4% ethanol solution only when compared with the ethanol/stress group (P≤0.05). On day 3, consumption of 4% ethanol solution of the ethanol/control group was greater than all other groups (P≤0.05). On day 5, the ethanol/control group consumed more 4% ethanol solution compared with the vehicle/control group and vehicle/stress group (P≤0.05). On days 7 and 9, the ethanol/stress group consumed more 4% ethanol solution when compared with the vehicle/stress group (P≤0.05).

Preference for 4% ethanol solution was altered in the ethanol/control group. Three-way ANOVA for preference for the 4% ethanol solution showed a significant effect of the ethanol factor (F_1,30_=6.52, P≤0.05). On day 1, the ethanol/control group had a higher preference for 4% ethanol solution when compared with the ethanol/stress group (P≤0.05). On day 3, the ethanol/control group had the greatest scores for preference of 4% ethanol solution (P≤0.05). On day 5, the preference of the ethanol/control group for 4% ethanol solution was higher only when compared with the vehicle/control group (P≤0.05). On day 7, the ethanol/stress group had higher preference for 4% ethanol solution than groups that did not receive ethanol (the vehicle/control group, and vehicle/stress group) (P≤0.05). No differences between groups were found for preference of 4% ethanol solution on day 9.

Animals in the ethanol/stress group consumed more 8% ethanol solution than animals in all other groups. Three-way ANOVA for consumption of 8% ethanol solution showed a significant effect of the ethanol factor (F_1,29_=6.77, P≤0.05) and a trend of significance for the interactions between stress and ethanol factors (F_1,29_=3.83, P=0.06). On day 1, the ethanol/stress group consumed significantly more 8% ethanol solution than the vehicle/control group and vehicle/stress group (P≤0.05). On day 3, the ethanol/stress group consumed more 8% ethanol solution only when compared with the vehicle/control group (P≤0.05). On day 5, the ethanol/stress group showed increased consumption of 8% ethanol solution compared with all experimental groups (P≤0.05). On days 7 and 9, the ethanol/stress group consumed more 8% ethanol solution compared with the ethanol/control group and vehicle/stress group (P≤0.05).

Preference for the 8% ethanol solution was also greater in animals of the ethanol/stress group. Three-way ANOVA showed a significant effect of the ethanol factor (F_1,29_=5.20, P≤0.05) and a trend of significance for the stress factor (F_1,29_=3.61, P=0.06). No changes were found in preference for 8% ethanol solution on day 1. On day 3, the preference for 8% ethanol solution of the ethanol/stress group was greater than the preference for 8% ethanol solution of the vehicle/control group and vehicle/stress group (P≤0.05). On days 5 and 7, the ethanol/stress group had higher preference for 8% ethanol solution than all other groups (P≤0.05).

Three-way ANOVA for fluid intake (water + ethanol 4% + ethanol 8%) showed a significant effect of the time factor (F_4,112_=7.3, P≤0.05). However, no differences were found between groups during the experiment on voluntary consumption of ethanol (data not shown).

## Discussion

Stress exposure is known to be an important contributory issue for alcohol abuse and alcoholism. However, the interaction between stress and alcohol-drinking behavior or alcohol withdrawal is complex and poorly understood.

Animals exposed to chronic consumption of ethanol over 3 weeks showed a decrease in locomotor activity in the OF during ethanol withdrawal, but no alterations in anxiety-like behaviors in the EPM. Contrary to our expectations, concomitant forced swimming stress did not aggravate anxiety-like behaviors related to ethanol withdrawal. Nevertheless, simultaneous exposure to ethanol and stress increased voluntary intake of ethanol (especially consumption of solutions containing high ethanol concentrations).

Ethanol withdrawal in animal models can be assessed through several behavioral and physiologic alterations after cessation of ethanol exposure. Protocols can vary according to the animal species, strains, time after cessation of ethanol consumption, and test apparatus employed (17). Despite the large body of data showing increased anxiety-like behaviors in EPM after cessation of ethanol intake (18), mainly in rats, ethanol withdrawal in mice is not always measured readily using this paradigm. Scholars have found it difficult to show increased anxiety-like behaviors in EPM during ethanol withdrawal (19,20). Those studies differ from each other and from our study according to mouse strain used and time point of withdrawal, thereby complicating comparisons between studies that could identify the reasons for different results.

Signs of ethanol withdrawal syndrome start to appear when levels of alcohol in blood approach zero. The type, duration and severity of these signs are dependent on the amount of ethanol administered and duration of treatment. In general, anxiety related to ethanol withdrawal appears 6-15 h after drug discontinuation (21). Our experiments were carried out during the light phase of the light-dark cycle, ∼7 h after lights had been turned on, and 12 h after removal of the liquid diet. This time does not represent the real time when mice stopped to consume ethanol solutions. Considering mice are more active and consume more food in the first hours of the dark cycle, they consumed more ethanol solution at this time period. Thus, in our study, anxiety-like behaviors related to ethanol withdrawal could have been assessed >12 h after removal of the liquid diet, when anxiety-like behaviors related to ethanol withdrawal may have disappeared.

In the OF apparatus, ethanol-treated mice showed decreased locomotor activity. Indeed, alterations in locomotor activity in the OF are, in general, used to indicate ethanol withdrawal. In rats, removal of ethanol could result in hyper-locomotion and hypo-locomotion whereas, in mice, hypo-locomotion is seen more commonly than hyper-locomotion (15,20,22 23 24).

Time spent at the center of the OF was increased in ethanol-treated animals. Despite use of this behavior in the measurement of anxiety in animal models (18), in our experiments it did not reflect an anxiolytic effect of ethanol withdrawal. When mice in ethanol-treated groups were put in the middle of the apparatus at the beginning of the OF procedure, they became immobile for a significant period of time, before their first move in the apparatus, a behavior that was very similar to “freezing”. Freezing is used largely as an index of fear in animal models (25). This result can be interpreted as an increase in fear behavior in ethanol-treated groups. Indeed, ethanol-treated animals spent more time in each central quadrant (Figure 3E) than vehicle-treated groups.

Psychomotor stimulation induced by drugs of abuse reflects activation of the dopaminergic mesolimbic pathway induced by these substances. This property seen in animal models is shared by all drugs known to cause addiction in humans (26). Similarly, locomotor activity seen during withdrawal of these drugs could reflect a “reward” pathway state. Withdrawal from the most commonly abused drugs causes hypo-locomotion and decreased basal activity in the dopamine reward pathway (27). In rats, an elevated intracranial self-stimulation reward threshold is found during withdrawal from chronic exposure to ethanol vapor, and is indicative of decreased sensitivity to reward in the brain (28). Extracellular dopamine levels in the nucleus acumbens are lower in ethanol-treated rats after cessation of ethanol exposure (29). Thus, we suggest that the decreased locomotor activity in OF during ethanol withdrawal reflects hypoactivity of the brain mesolimbic reward pathway of mice.

Exposure to stressful situations results in changes in locomotor activity and anxiety-like behavior in mice and rats. For example, chronic restraint stress increases locomotor activity (30) and could be anxiogenic in rats and mice (31,32). Our forced swimming stress procedure did not result in locomotor changes in EPM or OF. Animals exposed to ethanol and stress treatments did not show altered behavior compared with animals exposed only to ethanol treatment. Therefore, the stress protocol used in our work could not alter ethanol withdrawal. Forced swimming stress seems to be a weaker stressor when compared with other protocols. Bowers et al. showed lower corticosterone levels in rats exposed to forced swimming stress compared with restraint stressed rats (33).

Concomitant exposure to stress and ethanol results in complex interactions. For example, stress and ethanol alone cause impairment of memory function in object recognition tests and the y-maze apparatus. Concomitant stress/ethanol exposure reverses these effects. Alcohol also blocks stress-induced increases in anxiety-like behaviors in the plus maze test. However, alcohol cannot block weight loss and increases in plasmatic corticosterone levels induced by stress (13). In the brain, immobilization stress results in changes in catecholamine levels that are reversed by pre-stress ethanol administration (14). Thus, the interaction between stress and ethanol seems to be complex and specific for some behavioral alterations.

Data from experiments on voluntary consumption of ethanol showed that pre-exposure to ethanol caused an increase in the consumption of, and preference for, ethanol. This increase was observed only for 4% ethanol solution. Scholars have shown increased consumption of ethanol or self-administration of ethanol after forced and chronic pre-exposure to ethanol in rats and mice (34 35 36 37). The three-bottle choice used in our study allowed differentiation of ethanol preference according to the ethanol concentration in consumed solutions. Highly concentrated ethanol solutions have an unpleasant taste and are usually avoided by rodents. The increased intake of ethanol induced by chronic ethanol treatment seems to be specific for low-concentration solutions.

A large body of work examining the impact of acute or sub-chronic stress on ethanol consumption has been conducted on rodents. Several stress procedures have been employed and, despite a general observation that stress is associated with increased drinking of alcohol, the literature reveals equivocal findings: increased, decreased, or no change in alcohol consumption (38). Humans report that ethanol consumption alleviates symptoms of stress and anxiety (7). Exposure of humans to controlled stressful situations in laboratory settings increases ethanol intake (39). In our study, animals exposed to stress and a forced ethanol diet had increased consumption of, and preference for, ethanol during free-choice drinking. A comparison of voluntary consumption of different ethanol concentrations showed increased consumption and preference specifically for 8% ethanol solution (the most concentrated solution offered to the animals in our protocol). These results show that stressful situations during chronic exposure to ethanol can aggravate addiction-related behaviors by increasing drinking of beverages of high ethanol content.

Animal housing during testing of voluntary consumption of ethanol was probably not a significant stressor that would increase ethanol intake in mice because the effect of isolation stress in mice is significant only if isolation starts during early ages and not during adulthood (40). Nevertheless, we cannot definitively exclude that notion that individual housing may have acted as a mild stressor that could influence drinking behavior (particularly in the first days of the study).

The increase in ethanol intake in mice co-exposed to stress and ethanol in our study does not seem to be related to an attempt to alleviate the symptoms of ethanol withdrawal. First, the ethanol/stress group did not show higher levels of ethanol withdrawal compared with the ethanol/control group. Second, increased consumption of, and preference for, ethanol was long-lasting (continuing to appear on the last day of voluntary consumption of ethanol), whereas withdrawal (in general) was short-lived. Hence, concomitant exposure to ethanol and stress may result in long-lasting neurologic adaptations that aggravate ethanol addiction. The relationship between stress and ethanol drinking is complex because of many ethanol-related factors (e.g., history of use; pattern of drinking; timing of accessibility of ethanol relative to stress experience), along with stress-related factors (e.g., chronicity, type, intermittency, predictability) that interact with several biologic variables (e.g., age, sex, genetic background) (17).

In summary, chronic pre-exposure to ethanol led to a decrease in locomotor activity related to withdrawal of this substance, an increase in voluntary intake of ethanol, and preference for low-concentration ethanol solutions. Concomitant stress did not aggravate ethanol withdrawal, but potentiated the intake of and preference for ethanol, thereby changing patterns of voluntary consumption towards highly concentrated ethanol solutions.
